# Prognostic Value and Genome Signature of m6A/m5C Regulated Genes in Early-Stage Lung Adenocarcinoma

**DOI:** 10.3390/ijms24076520

**Published:** 2023-03-30

**Authors:** Long Tian, Yan Wang, Jie Tian, Wenpeng Song, Lu Li, Guowei Che

**Affiliations:** 1 Lung Cancer Center, Department of Thoracic Surgery, West China Hospital, Sichuan University, Chengdu 610041, China; 2 Lung Cancer Center, West China Hospital, Sichuan University, Chengdu 610041, China

**Keywords:** early-stage LUAD, m6A, m5C, prognosis, genome feature

## Abstract

RNA modifications implicate pathological and prognosis significance in cancer development and progression, of which, m6A and m5C are representative regulators. These RNA modifications could produce effects on the function of other RNA by regulating gene expression. Thus, in this study, we aimed to explore the correlation between m6A/m5C regulators and early-stage lung adenocarcinoma (LUAD). Only the early-stage LUAD samples were included in this investigation, and the RNA-seq dataset of The Cancer Genome Atlas (TCGA) cohort was utilized to evaluate the expression of 37 m6A/m5C regulated genes. Based on the expression level of these 37 genes, early-stage LUAD patients were divided into 2 clusters, which were performed by consensus clustering, and the m6A/m5C subtypes had significantly different prognostic outcomes (*p* < 0.001). Cluster1, which has a better prognosis, was characterized by the C3 (inflammatory) immune subtype, low immune infiltration, chemokine expression, major histocompatibility complex (MHC) expression, and immune checkpoint molecule expression. Furthermore, compared with cluster1, cluster2 showed a T cell exhaustion state, characterized by a high expression of immune checkpoint genes, and immune cells, such as T cells, CD8+ T cells, cytotoxic lymphocytes, NK cells, and so on. In addition, patients in cluster2 were with high tumor mutational burden (TMB) and numerous significant mutated oncogene and tumor suppressor genes, such as WNT10B, ERBB4, SMARCA4, TP53, and CDKN2A (*p* < 0.001). A total of 19 genes were mostly related to the prognosis of LUAD and were upregulated in cluster2 (*p* < 0.05), showing a positive correlation with the mRNA expression of 37 m6A/m5C regulated genes. The predictive risk model was constructed using Cox and LASSO (least absolute shrinkage and selection operator) regression analysis. Finally, a seven-gene m6A/m5C risk model, comprising of METTL3, NPLOC4, RBM15, YTHDF1, IGF2BP1, NSUN3, and NSUN7, was constructed to stratify the prognosis of early-stage LUAD (*p* = 0.0049, AUC = 0.791). The high-risk score was associated with a poorer prognosis. This model was also validated using two additional GEO datasets: GSE72094 (*p* = 0.011, AUC = 0.736) and GSE50081 (*p* = 0.012, AUC = 0.628). In summary, it was established that the m6A/m5C-regulated genes performed a crosstalk function in the mRNA expression of early-stage LUAD. By interacting with other mRNA genes, m6A/m5C modification disturbs DNA replication and the tumor immune microenvironment (TIME). The seven-gene risk model may be a critical tool for the prognostic assessment of early-stage LUAD.

## 1. Introduction

According to global cancer data, lung cancer is the leading cause of cancer-related death [1]. Among them, non-small cell lung cancer (NSCLC) accounts for approximately 85% of lung cancer cases based on histological classification, and the remaining 15% are small cell lung cancer (SCLC). Lung adenocarcinoma (LUAD) and lung squamous cell carcinoma (LUSC) are the most common subtypes of NSCLC [2]. The 5-year survival rate for lung cancer patients ranges from 92% for IA1 to 53% for IIB, although targeted therapy and immunotherapy have changed the lung cancer treatment landscape. The main reason is the minor benefit group and postoperative micrometastasis [3]. It is urgent to explore new prognostic biomarkers and drug targets to improve early-stage lung cancer treatment efficiency.

Recently, abundant studies have confirmed the implications of RNA modifications in human cancer [4,5]. For instance, N6-Methyladenosine (m6A), methylated adenosine at the N6 position, has emerged as an essential internal RNA modification in the expression of messenger RNA (mRNA) and non-coding RNAs (ncRNAs) in most eukaryotic species [6]. Shen et al., (2022) reported that IGF2BP1 was a significantly highly expressed gene in NSCLC and could stabilize TK1 expression via m6A modification and promote tumor cell proliferation, migration, and invasion [7].

The 5-methylcytosine (m5C) could predict prognosis risk and influence the immune microenvironment of lung squamous cell carcinoma (LUSC) [8]. Bai, M. and C. Sun found that m5C methyltransferase could affect clinical prognosis by regulating the expression of three lncRNAs in LUAD [9]. NSUN2, an RNA methyltransferase, has been reported to be highly expressed in pan-cancer and displays different clinical values in various cancers [10]. However, few studies have reported on the correlation between m6A and m5C regulation. Barbieri, I. and T. Kouzarides systematically reviewed the roles of RNA modifications in cancers, respectively, and proposed that some RNA methyltransferases share a common structure with DNA and protein methyltransferases [4]. For instance, the m6A methyltransferase enzyme complex METTL3-METTL14 and m5C writer NSUN2 belong to the same Rossmann fold family of methyltransferases, which suggests a potential link in structure and function between m6A and m5C modifications. 

Similar to DNA and proteins, RNA may be methylated and demethylated by methyltransferases (“writers”) and demethylases (“erasers”) and recognized by reading proteins (“readers”). RNA modification could have an influence on the function of other RNA by interacting with microRNA (miRNA) and long ncRNA (lncRNA), which could then have a positive or negative impact on cancer development [4]. Lee et al. summarized the functional crosstalk between mRNA modifications, and the m6A and m5C regulation that occurred in the exact mRNA location of the CDKN1A gene and argued that this could promote the translation of CDKN1A. These dynamic crosstalks may be necessary for stabilizing cancer-related events [11]. 

This study concentrates on two previously investigated RNA modifications, m6A, and m5C, and explores the significant prognosis value in early-stage LUAD. The Cancer Genome Atlas (TCGA) dataset was used to analyze the expression level of 37 m6A and m5C-related genes in early-stage LUAD patients. The prognosis value of m6A and m5C was revealed according to bioinformatics analysis based on the TCGA and GEO datasets. The risk model was constructed to evaluate prognosis and precisely stratify early-stage LUAD patients.

## 2. Results

### 2.1. Identification of Molecular Clusters of Early-Stage LUAD Based on m6A/m5C-Related Genes

**Figure 1:**
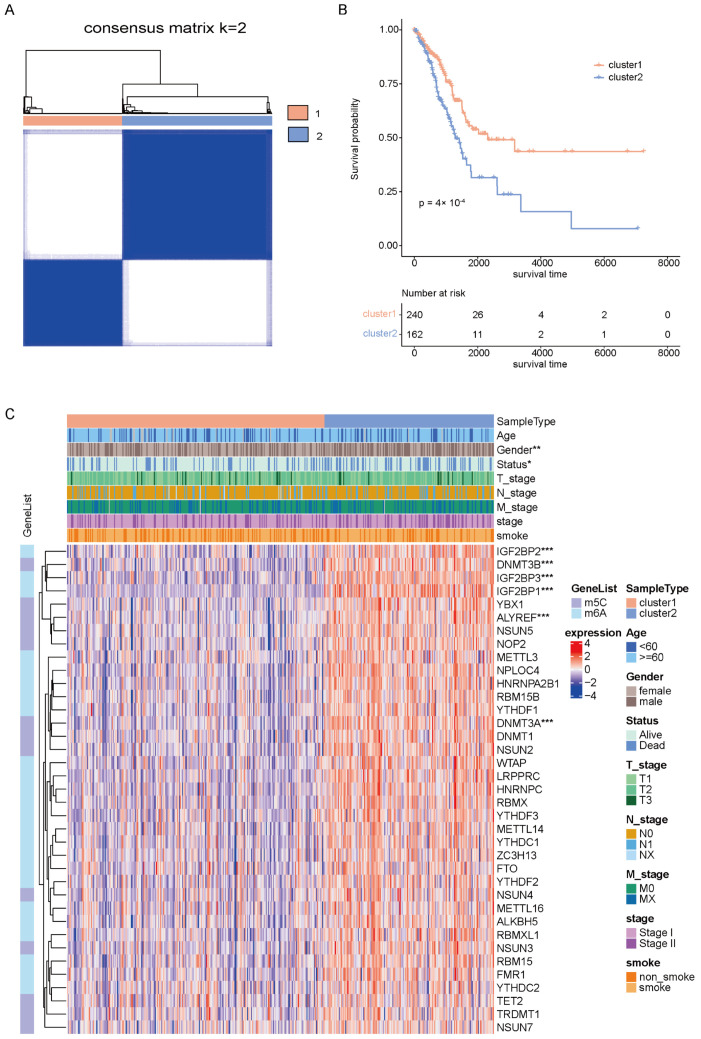
Consensus clustering of m6A/m5C-related genes in early-stage LUAD. (**A**) Consensus clustering shows that two clusters are the most stable clusters. (**B**) Kaplan-Meier curves for OS of the early-stage TCGA-LUAD cohort with m6A/m5C subtypes. (**C**) Unsupervised clustering of all m6A/m5C-related genes in early-stage TCGA-LUAD cohorts. m6A/m5C cluster, age, gender, status, T stage, M stage, N stage, stage, and smoke were used as patient annotations. *, *p* < 0.05; **, *p* < 0.01; ***, *p* < 0.001.

TCGA-LUAD early-stage patients were used for the following study. Based on the mRNA expression of 37 m6A/m5C regulatory genes, the unsupervised clustering method was utilized for the subtype analysis of early-stage LUAD patients. We chose two as the optimal k value, and the 402 patients were divided into cluster1 (N = 240) and cluster2 (N = 162) (Figure 1A and Figure S1A). In addition, when visualizing the results of the PCA algorithm, we found that the clustering discrimination effect was excellent (Figure S1B), which further confirmed two remarkably different subtypes of early-stage LUAD. K-M survival analysis showed a significant difference in OS between the two m6A/m5C-related clusters, and the patients in cluster1 demonstrated a significantly better OS than those in cluster2 (log-rank test *p* < 0.05) (Figure 1B). The expression of m6A/m5C-related genes in two clusters and a correlation with clinical characteristics are shown in Figure 1C. Most genes were highly expressed in cluster2, and gender and stage characteristics were significantly different in the two clusters. Moreover, there was a statistically significant gender difference (*p* < 0.01) and survival status (*p* < 0.05) between the two early-stage LUAD subtypes (Figure S1C–F).

### 2.2. Immune Microenvironments of m6A/m5C Subtype in Early-Stage LUAD

**Figure 2:**
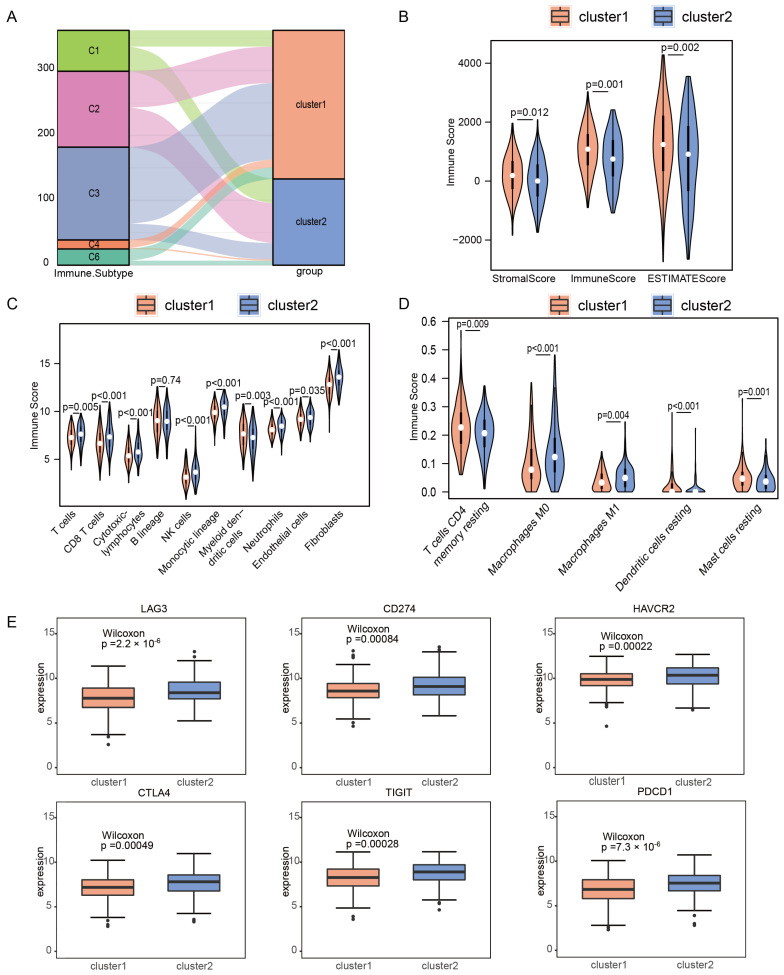
Immune landscape of m6A/m5C subtypes in the early-stage TCGA-LUAD cohort. (**A**) Comparison between the m6A/m5C subtypes established in this study and immune subtypes in existing TCGA cancers. (**B**) Stromal score, immune score and estimate score between the m6A/m5C subtypes. (**C**) Comparison of immune scores calculated using MCPcounter between the m6A/m5C subtypes. (**D**) Comparison of immune scores calculated using CIBERSORT between the m6A/m5C subtypes. (**E**) The expression of immune checkpoint molecules in m6A/m5C subtypes.

Thorsson et al. identified six immune subtypes for pan-cancers, including C1 (wound healing), C2 (IFN-γdominant), C3 (inflammatory), C4 (lymphocyte depleted), C5 (immunologically quiet), and C6 (TGF-β dominant) [12]. A further comparison of immune subtype features between the two m6A/m5C subtypes showed that cluster2 had a high proportion of the C1 subtype and a low proportion of the C3, C4, and C6 subtypes, which were associated with poor prognosis (Figure 2A). To explore the difference in immune characteristics between the two m6A/m5C-related clusters, we then analyzed the heterogeneity of immune cell infiltration based on Estimate, MCPcounter, and CIBERSORT algorithms. The results showed a significant difference in immune scores between the two groups. The immune score of cluster1 was higher than that of cluster2 (*p* = 0.00061). In addition, the stromal score of cluster2 was lower than that of cluster1 (*p* = 0.01172, Figure 2B). There were notable differences in the proportion of immune cells between cluster1 and cluster2. Compared with cluster1, cluster2 demonstrated an increased expression of T cells, CD8+ T cells, cytotoxic lymphocytes, NK cells, monocytic lineage cells, neutrophils, endothelial cells, fibroblasts, and macrophages M0 and M1. In addition, cluster2 showed a lower infiltration of myeloid dendritic cells, T cells CD4 memory resting, and Mast cell resting (Figure 2C,D). As shown in Figure 2E, cluster2 exhibited a higher expression of six immune checkpoint genes, indicating a tendency for immune escape. Subsequently, we also explored the different chemokines gene expressions between the two clusters. Among the 41 chemokines, 25 showed statistically significant differences between the 2 subtypes (Figure S2A), and 11 of 18 chemokine receptor genes were expressed differently between the 2 m6A/m5C-related clusters (Figure S2C). Interestingly, most chemokine and chemokine receptor genes were expressed higher in cluster2. Cytolytic activity (CYT) showed that CYT in cluster2 was significantly higher than in cluster1 (Figure S2B). At the same time, we studied the relationship between the two subtypes and major histocompatibility complexes and HLA family genes. The expression levels of 14 major histocompatibility complexes and HLA family genes, including HLA-A and HLA-B, were relatively high in cluster2 (Figure S2D,E).

### 2.3. Somatic Alteration Landscape of Patients with Early-Stage LUAD in Different Clusters

**Figure 3:**
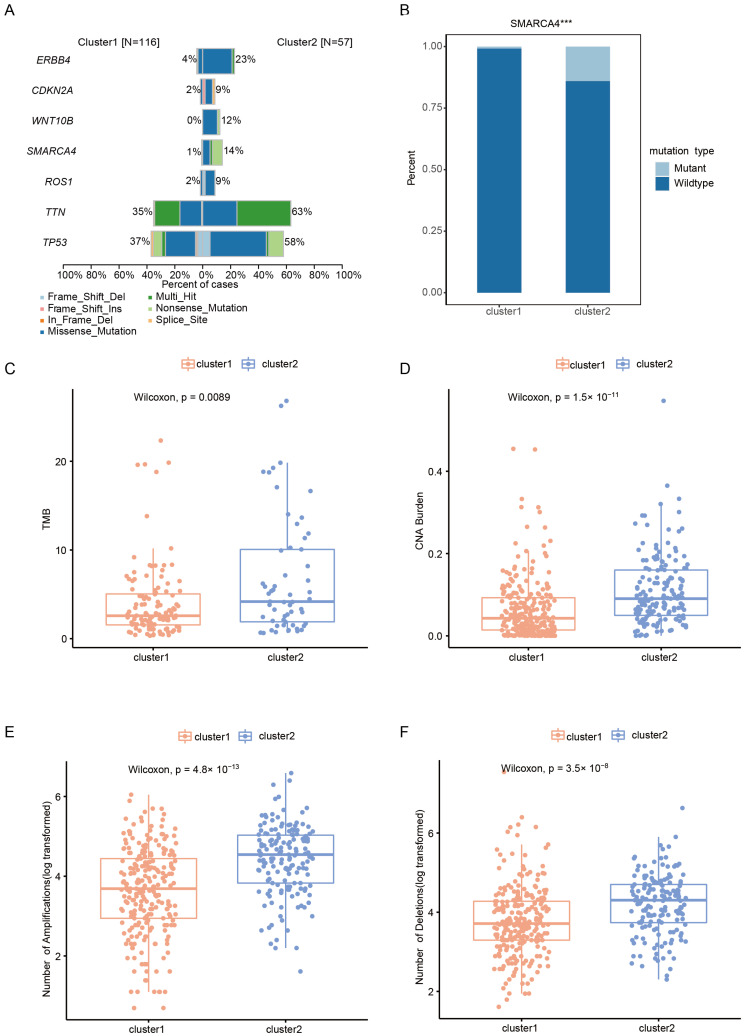
Somatic variations of the m6A/m5C clusters. (**A**) Barplots showing the seven differential mutated genes of clusters. (**B**) Bar plots showing the mutation frequency of SMARCA4 in the clusters. (**C**) Comparisons of TMB between two clusters. (**D**) Comparisons of the CNA burden between two clusters. (**E**) Comparisons of the number of amplifications between two clusters. (**F**) Comparisons of the number of deletions between two clusters. ***, *p* < 0.001.

The somatic mutations were analyzed with the TCGA early-stage LUAD dataset, and waterfall maps were created to describe the top 20 mutant genes in the 2 clusters. The most frequently occurring mutated genes in cluster1 and cluster2 were TP53 and TTN, respectively (Figure S3A,B). Among these somatic alterations, 436 genes had significantly different mutation frequencies between the 2 clusters (*p* < 0.05). In addition, for the commonly mutated oncogene and tumor suppressor genes of LUAD, the mutation frequencies SMARCA4, ERBB4, CDKN2A, WNT10B, ROS1, TTN, and TP53 were higher in cluster2 (Figure 3A,B and Figure S3C–H). The tumor mutational burden (TMB) of cluster1 was lower than that of cluster2 (*p* = 0.00089) (Figure 3C). In terms of CNA, compared with cluster1, cluster2 expressed more CNA burdens, amplifications, and deletion (Figure 3D–F).

Mutation signature analysis showed that early-stage LUAD was associated with three signatures (Figure S4A). Signature_1 was related to APOBEC cytidine deaminase (COSMIC_13). Signature_2 was similar to DNA mismatch repair (COSMIC_6), which occurs in microsatellite unstable tumors and is associated with the loss of DNA mismatch repair. Signature_3 was related to smoking (COSMIC_4) (Figure S4B). The fraction of three signatures were obviously different between the two clusters (Figure S4C,D).

### 2.4. DEGs Identification and Functional Analysis between Clusters

**Figure 4:**
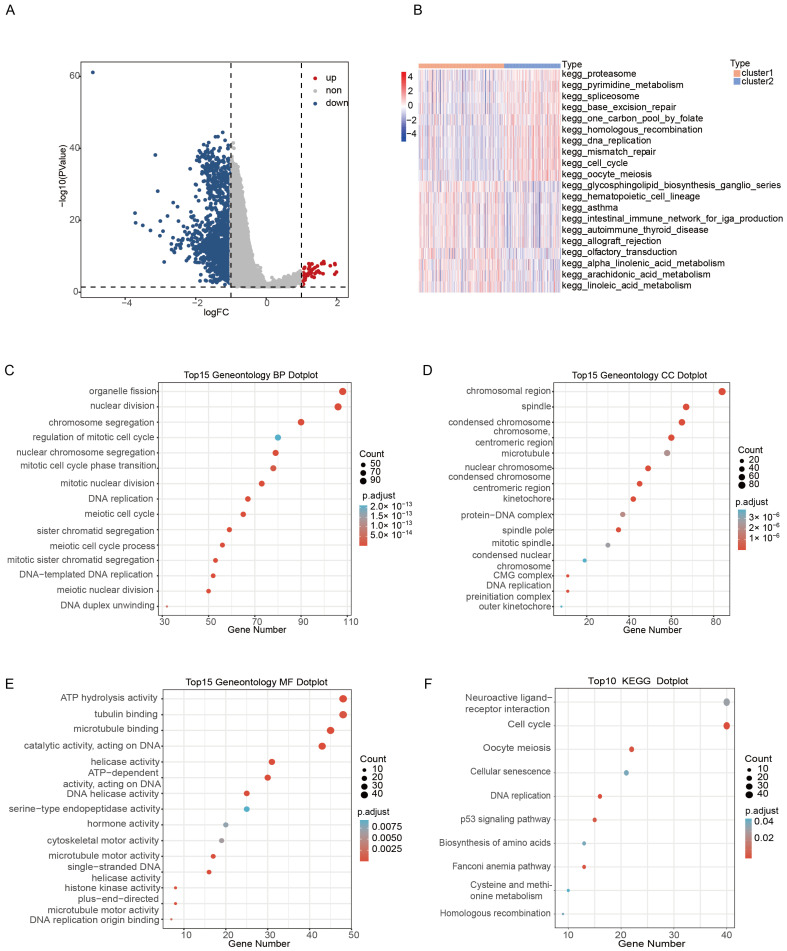
Difference analysis and functional analysis between m6A/m5C subtypes. (**A**) Volcano map of DEGs between the m6A/m5C subtypes. (**B**) The 20 biological pathways of two m6A/m5C subtypes by GSVA. Red represents the activation of biological pathways and blue represents the inhibition of biological pathways. (**C**) BP results of DEGs in m6A/m5C subtypes. (**D**) CC results of DEGs in m6A/m5C subtypes. (**E**) MF results of DEGs in m6A/m5C subtypes. (**F**) KEGG results of DEGs in m6A/m5C subtypes.

A total of 1625 DEGs were selected between cluster1 and cluster2 following the threshold of *p* < 0.05 and |log2FC| > 1, of which 51 genes were upregulated; 1574 genes were down-regulated (Figure 4A). In addition, GO annotation analysis showed that DEGs were enriched in 295 biological processes, such as nuclear division and chromosome segregation, 56 cellular components, including the chromosomal region and condensed chromosome, and 22 molecular functions, for example, single-stranded DNA helicase activity and DNA helicase activity (*p*.adjust < 0.05). The top 15 annotations are shown in Figure 4C–E), and the original analysis statistic of the GO annotation is listed in Table S4. In addition, KEGG enrichment analysis showed that DEGs were involved in 315 pathways (*p*.adjust < 0.05), including the Fanconi anemia pathway, p53 signaling pathway, homologous recombination, and other pathways related to the tumor (Figure 4F). The KEGG enrichment analysis results for the two clusters are shown in Table S5.

To determine the difference between the 2 m6A/m5C-related cluster activation pathways, we performed GSVA with MSigDB v7.5 and found that the 2 subtypes showed 72 different pathways (*p*.adjust < 0.01). The detailed GSVA enrichment results are shown in Table S6. The heatmap showed that cluster1 was significantly enriched in the intestinal immune network for iga production, the glycosphingolipid biosynthesis ganglio series, and so on, while cluster2 was enriched in DNA replication, mismatch repair, homologous recombination, and so on (Figure 4B). At the same time, GSEA analysis confirmed differences in 52 pathways between the 2 m6A/m5C clusters (*p*.adjust < 0.05, Figure S5).

### 2.5. Identification Cluster2 Specific Hub Gene

**Figure 5:**
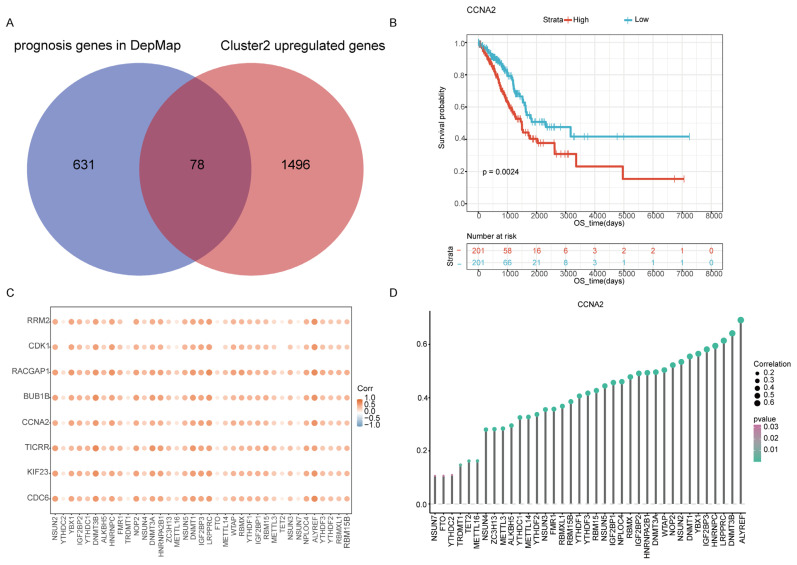
Screening of prognostic markers for early LUAD. (**A**) Venn diagram showing the intersection of cluster2 upregulated genes and the genes which are essential to LUAD human cell line growth using the DepMap database. (**B**) Kaplan–Meier analysis showing the association between the expression of CCNA2 and early-stage LUAD patient overall survival (OS) for the TCGA cohort. (**C**) Correlation between nine survival-related genes and m6A/m5C regulatory genes. (**D**) Correlation between CCNA2 and m6A/m5C regulatory genes.

We found that 709 genes performed essential roles in the survival of LUAD cell lines in the Dep map database. Among the 709 candidate genes, 78 were relatively highly expressed in cluster2 (Figure 5A). Then, we explored survival based on the expression of these 78 genes, and the survival analysis showed that 9 genes (CCNA2, CDC6, RACGAP1, SGOL1, TICRR, RRM2, BUB1B, KIF23, and CDK1) had prognostic significance in TCGA and GEO databases (GSE72094 and GSE50081). In patients with early-stage LUAD, the high expression level of these nine genes was associated with poor prognosis in TCGA-LUAD, GSE72094, and GSE50081 separately. (Figure 5B and Figures S6–S8). Additionally, these nine genes were significantly positively correlated with most of the m6A/m5C-related regulatory genes (Figure 5C,D).

### 2.6. Construction of the m6A/m5C Related Prognostic Model

**Figure 6:**
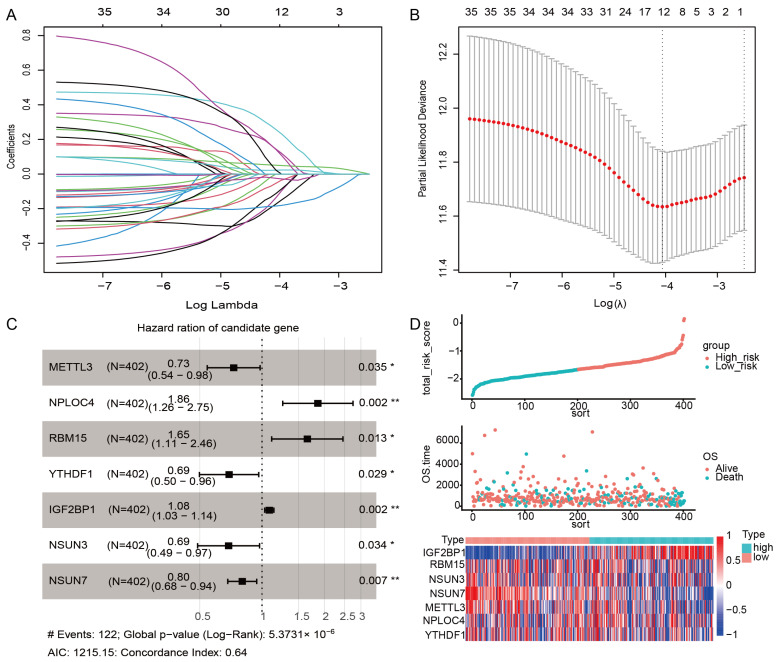
Construction of the m6A/m5C-related prognostic model. (**A**) The trajectory of 37 m6A/m5C regulatory genes, wherein the horizontal axis represents the log value of m6A/m5C regulatory genes lambda, and the vertical axis represents the coefficient of m6A/m5C regulatory genes. (**B**) LASSO coefficient profiles of 11 selected m6A/m5C regulatory genes in a 10-fold cross-validation. The vertical dotted lines were drawn at the optimal values by using the minimum criteria and 1-SE criteria. (**C**) The forest plot of the associations between the expression of seven prognostic molecules and OS in the early-stage TCGA-LUAD training cohort. (**D**) The risk score, survival status, and the expression of seven genes in early-stage TCGA-LUAD training set. *, *p* < 0.05; **, *p* < 0.01; #, footnote for the figure.

Early-stage LUAD patients with OS information in the TCGA cohort were used to establish predictive models. The best prognostic signatures were screened from 37 m6A/m5C-related regulatory genes by LASSO-Cox regression (Figure 6A,B) and multivariate Cox analysis (Figure 6C). Finally, a prognostic model based on seven m6A/m5C-related regulatory genes (METTL3, NPLOC4, RBM15, YTHDF1, IGF2BP1, NSUN3, and NSUN7) was constructed. The final signature score formula for the seven genes was:

RiskScore = −0.152 × METTL3 + 0.168 × NPLOC4 + 0.138×RBM15 − 0.079 ×  YTHDF1 + 0.074 × IGF2BP1 − 0.103 × NSUN3 − 0.216 × NSUN7.

The risk score of each sample was calculated according to the gene expression, and the risk score distribution of the sample was drawn (Figure 6D). Furthermore, to assess the expression of seven m6A/m5C-related regulatory genes in terms of the protein level, we elicited immunohistochemical images, taking advantage of the HPA database. From Figure S9, it can be intuitively seen that the protein expression of 5 m6A/m5C-related regulatory genes was significantly higher in tumor tissues than in normal tissues. 

**Figure 7:**
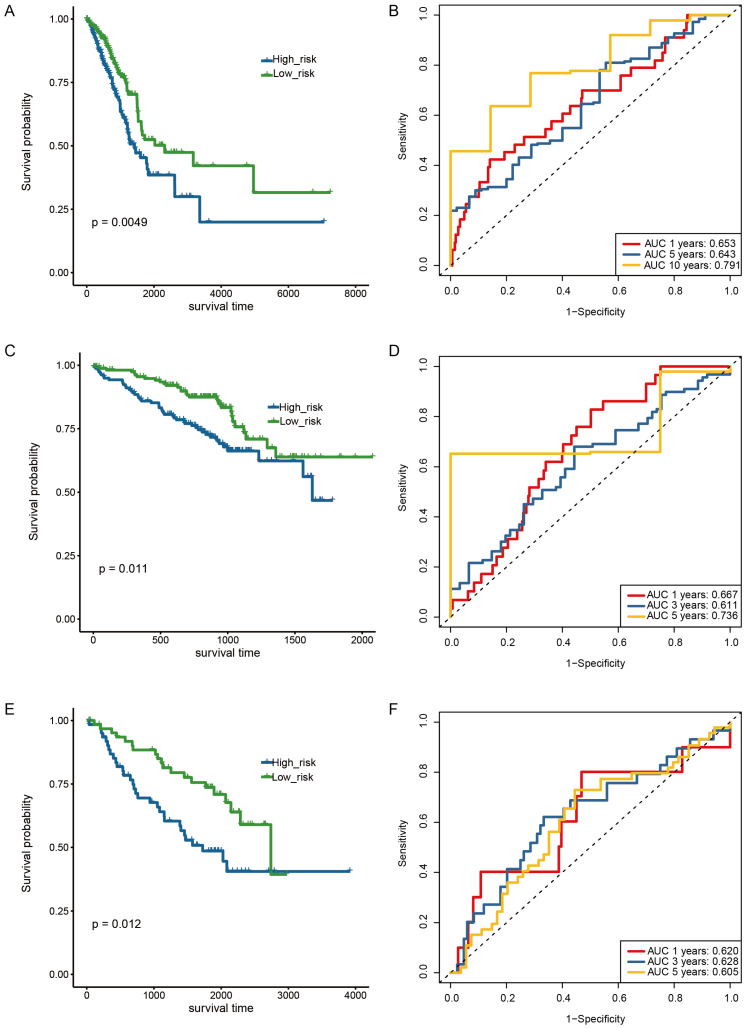
Validation of the seven genes signature in the training set and test sets. (**A**) ROC curve and AUC of the seven-gene signature in early-stage TCGA-LUAD set. (**B**) KM curve demonstrating survival predicted by the seven genes signature in the early-stage TCGA-LUAD set. (**C**) ROC curve and AUC of the seven genes signature in GSE72094. (**D**) KM curve demonstrating survival predicted by the seven genes signature in GSE72094. (**E**) ROC curve and the AUC of seven genes signature in GSE50081. (**F**) KM curve demonstrating survival predicted by the seven genes signature in GSE50081.

Patients in the TCGA dataset were divided into high-risk and low-risk groups according to their risk scores. Kaplan–Meier (KM) survival analysis was performed on training and test data sets to evaluate the predictive ability of m6A/m5C-related signature scores. In the TCGA dataset, high-risk patients showed worse OS than low-risk patients (*p* = 0.0049, Figure 7A). Similar results were observed in the test dataset (GSE72094 and GSE50081) (Figure 7C,E). Meanwhile, time-dependent AUC showed that the m6A/m5C-related signature scores were significant for predicting OS in patients with early-stage LUAD in TCGA and GEO datasets. The time-dependent AUC values of TCGA for predicting prognosis at 1-, 5-and 10-year was 0.653, 0.643, and 0.791 (Figure 7B). As for the predictions on the GSE72094, the areas under the ROC curve of OS in 1-, 3-, and 5-year were 0.667, 0.611, and 0.736, respectively (Figure 7D). In addition, the time-dependent AUC values in GSE50081 for 1-, 3- and 5-year survival were 0.620, 0.628, and 0.605, respectively (Figure 7F).

**Figure 8:**
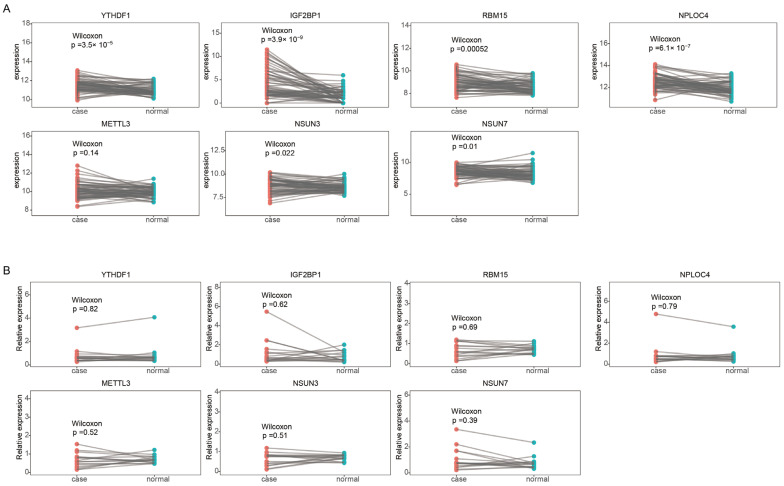
The mRNA expression of seven risk genes. (**A**) The different mRNA expression of seven risk genes between paired tumor and normal samples in early-stage TCGA-LUAD. (**B**) RT-qPCR analyses seven risk genes expression in paired tumor tissues and paracancerous tissues of LUAD.

Furthermore, the different mRNA expressions of the above seven risk genes between paired tumor and normal samples in TCGA-LUAD datasets were analyzed, which showed an obviously upregulated expression in early-stage LUAD (Figure 8A). To further explore the mRNA expression level of 7 m6A/m5C regulated genes, qRT-PCR experiments were implemented utilizing 16 paired frozen fresh tumor tissues and paracancerous tissues of LUAD, and the primer sequences are presented in Table 1. Although there was no significant difference in the expression of these seven genes between normal and tumor tissues, the mRNA expression levels of seven genes in LUAD were relatively higher than those in normal human lung cells, which was consistent with the TCGA results (Figure 8B). The 2^−(∆∆Ct)^ value of RT-qPCR experiment for 16 pairs of tumor and normal samples from LUAD patients are shown in Table S7.

## 3. Discussion

Recently, numerous biomarkers, including EGFR, ALK, KRAS, TP53, and others, have provided NSCLC patients with important advantages in a broad range of clinical decisions, such as target therapy, immunotherapy, and prognosis evaluation, and have also strengthened our recognition of the biological properties and functions of NSCLC [13,14]. Some studies have discovered that variations in gene expression influenced the proliferation and metastasis of lung cancer cells. Zhong et al. reported that overexpressed AFAP1-AS1 promotes lung cancer cell migration and invasion by upregulating c-Myc [15]. Given the complexity and heterogeneity of cancer, it is necessary to continuously investigate molecular indicators that are linked to cancer prognosis based on gene expression patterns to identify additional therapeutic targets. 

RNA methylations are representative RNA post-transcriptional modifications that are closely related to the onset and progression of malignancies [16]. For example, the overexpression of m6A methyltransferase METTL3 could regulate the expression of the JUNB gene, which contributed to the epithelial-mesenchymal transition (EMT) in lung cancer [17]. YTHDF2 was significantly upregulated in lung cancer, which could promote and enhance tumor growth by facilitating the 6GPD mRNA translation [18]. Prior to the study, we investigated the prognosis value of four commonly occurring RNA methylation modification regulators, including m6A, m5C, m7G, and m1A-regulated genes. Finally, only the expression of some m6A and m5C regulated genes were confirmed highly correlated with the prognosis of early-stage LUAD. Thus, we explored the prognosis significance of 37 m6A and m5C-regulated genes in early-stage LUAD, and 7 out of the 37 m6A/m5C-regulated genes (METTL3, NPLOC4, RBM15, YTHDF1, IGF2BP1, NSUN3, and NSUN7) were ultimately utilized to establish the prediction model.

According to bioinformatics analysis, early-stage LUAD could be divided into two subtypes based on the mRNA expression of 37 m6A/m5C-related genes, which showed significantly different survival times between them. Cluster1 showed a higher survival advantage when compared with cluster2, and the clinical characters revealed that more male and deceased patients were enriched in cluster2. Paggi et al. reviewed gender-related disparities in NSCLC and proposed that the higher mortality rates in male patients for lung cancer were associated with smoking habits and lifestyle rather than with gender differences [19]. To further investigate the causes of poor survival in cluster2, immune cell infiltration, somatic mutations, and KEGG pathway enrichment were conducted subsequently. Cluster1 was characterized by a predominately inflammatory immune subtype (C3) and downregulated M1 macrophage cells infiltration. Thorsson, V. et al. demonstrated that the C3 immune subtype had the best prognosis and fewer alterations in immune genes, which is consistent with our observation of significantly downregulated M1 macrophages and immune checkpoint molecule genes, such as PD-(L)1, CTLA4, and LAG3 [12]. These distinct immune cell expressions between two subtypes may be due to the modulation of TME by m6A/m5C epigenetic modification. The highly elevated expression of immune checkpoint molecular genes may reflect a suppressed immune state in cluster2. The m6A/m5C modification could regulate the immune checkpoint molecular expression and affect the activation of antitumor immune cells. Taken together, the above immune features in Cluster2 exhibited a T cells exhaustion state alongside the loss of the effector function, characterized by a high expression of immune checkpoint genes, chemokine and chemokine receptor genes, and immune cells, such as T cells, CD8+ T cells, cytotoxic lymphocytes, NK cells, and so on [20,21]. In addition, some studies found that epigenetic modification changes were involved in the exhaustion progress, which implied the correlation between m6A/m5C epigenetic modification genes and the loss of the immune effector, which contributed to poor survival outcomes [22]. Fortunately, substantial studies have identified that by block immune checkpoint pathway could reverse T cell exhaustion in cancers, which suggested that the patients in cluster2 might benefit from immunotherapy [21]. 

Furthermore, synchronized TCGA mutation data also demonstrated distinct genomic alterations between the two subtypes. Interestingly, the mutation frequency of some recurrently oncogenic or suppressor genes, such as WNT10B, ERBB4, TTN, SMARCA4, TP53, and CDKN2A, was significantly different between the two subgroups, and these mutations occurred more commonly in the poor survival subgroup. Lou et al. found upregulated YTHDF1 expression in KRAS and TP53 mutant NSCLC cell lines, which could promote Cyclin B1 Translation through m6A modulation and contribute to the poor prognosis of KRAS/TP53 co-mutation LUADs [23]. This further implied that m6A regulation might be involved in the occurrence of oncogene mutations and impacting signal transduction pathway. Pathway enrichment analysis further confirmed the significantly activated DNA replication, mismatch repair, and homologous recombination pathway contributed to the genome instability and poor prognosis of cluster2. This verified that m6A and m5C epigenetic modification could affect the prognosis of early-stage LUAD with specific oncogenic or suppressor gene mutation by modulating mRNA expression, which may offer new perspectives for cancer therapy. 

Subsequently, we identified the 9 significantly different expression genes (CCNA2, CDC6, RACGAP1, SGOL1, TICRR, RRM2, BUB1B, KIF23, and CDK1) between the 2 subtypes, and verified that these genes were positively related to 37 m6A/m5C-related genes. Wu et al. confirmed that m6A demethylase FTO could modulate adipogenesis metabolism by regulating the cell cycle via m6A-dependent and YTHDF2-mediated mechanisms, which may promote cancer development [24]. Yang et al. found that the LCAT1/IGF2BP2 complex increased the expression of CDC6 by regulating the mRNA in an m6A manner, which helped to promote the growth and migration of lung cancer cells [25]. Therefore, m6A/m5C epigenetic modification may regulate the gene expression of these nine genes by modulating their mRNA methylation, which in turn could affect the tumor progression and prognosis of early-stage LUAD. However, these results need to be validated by in-depth biological experiments in vivo and in vitro. 

Finally, a seven m6A/m5C gene (METTL3, NPLOC4, RBM15, YTHDF1, IGF2BP1, NSUN3, and NSUN7) prognostic risk model was constructed to precisely stratify early-stage LUAD patients, and the high-risk score suggested a poor prognosis for early-stage LUAD, which could be well applied in the clinical evaluation and therapy strategies. These seven genes were significantly overexpressed in LUAD, as evidenced by the RT-qPCR experiment validation using the paired tumor tissues and paracancerous tissues of LUAD. The enhanced protein expression of these seven genes in lung cancer compared to normal lung tissues further confirms the specific regulation of these seven genes in lung cancer. Overall, the above investigation indicated that risk models have a robust prognosis value and prediction ability.

Nevertheless, this study still has some limitations. This study mainly focused on the prognostic value of m6A/m5C mRNA regulation, but more prospective studies based on in vitro and vivo validation are required to thoroughly elucidate the biological function of m6A/m5C modification in LUAD.

Conclusively, m6A/m5C methylation modification could regulate the expression of vital prognosis genes and oncogene alterations in early-stage LUAD. The m6A/m5C regulated genes may be involved in the progression of T cell exhaustion and signal transduction pathways, such as DNA replication and mismatch repair, which could further contribute to the poor prognosis and progression of early-stage LUAD. Given that the regulation of m6A/m5C-regulated genes have been identified as an important prognostic biomarker in early-stage LUAD, investigating therapeutic drugs that can target m6A/m5C may be a promising approach. RNA modification proteins could be ideal therapy targets, and biological structures are currently available for most enzymes, enabling convenient drug development. In conclusion, m6A/m5C regulators may become a potential therapeutic target and predictive biomarker for early-stage LUAD, which need more in depth clinical studies to validate in future.

## 4. Materials and Methods

### 4.1. Publicly Available Cohort Datasets and Preprocessing

The 37 writer, reader, and eraser genes of m6A and m5C were obtained from the literature [4,26]. The gene expression profiles and corresponding clinical information of patients with LUAD cohort (TCGA-LUAD, GSE72094, and GSE50081) in The Cancer Genome Atlas (TCGA, TCGA-LUAD (https://gdc-hub.s3.us-east-1.amazonaws.com/download/TCGA-LUAD.htseq_counts.tsv.gz, accessed on 3 August 2022) [27] and Gene Expression Omnibus (GEO, GSE72094 (https://www.ncbi.nlm.nih.gov/geo/query/acc.cgi?acc=GSE72094, accessed on 8 August 2022; GSE50081 (https://www.ncbi.nlm.nih.gov/geo/query/acc.cgi?acc=GSE50081, accessed on 1 August 2022) [28] were analyzed. Patients with stages III and IV were excluded. The patients’ detailed clinical characteristics and correlation analysis for the TCGA-LUAD, GSE72094, and GSE50081 cohorts are listed in Tables S1–S3 separately. The TCGA RNA-Seq data, clinical data, and copy number variation data were downloaded from the UCSC Cancer Browser (XENA, https://xenabrowser.net/datapages/, accessed on 3 August 2022). As for the dataset from GEO, the corresponding platform annotation files were obtained to convert the probes into gene symbols. The average expression level of multiple probes corresponding to the same gene symbol was taken as the expression level of the corresponding genes. 

### 4.2. Consensus Clustering Analysis of m6A/m5C Regulated Gene

We identified 402 patients with pathology stage I and II as the early-stage LUAD cohort. These 402 cases of early-stage LUAD were clustered and screened according to the expression of m6A/m5C regulatory genes by the ConensusClusterPlus package [29]. The Euclidean distance was used to calculate the similar distance between samples, and the K-means were used for clustering. The clustering was performed using 1000 iterations to ensure classification stability, and each iteration contained 80% of the samples. Based on the optimal K value, the patients of early-stage LUAD in TCGA were divided into cluster1 and cluster2. The results of the principal component analysis (PCA) were plotted to show the difference between the two clusters. The Kaplan–Meier (K–M) survival analysis was conducted to assess the differences between the two clusters’ overall survival (OS). A log-rank test was used to estimate the relationship between m6A/m5C subtypes and clinicopathological features, such as age, gender, T stage, and smoke. A heatmap was plotted to reveal the expression of m6A/m5C regulatory genes in the two m6A/m5C subtypes through the ‘ComplexHeatmap’ R package [30].

### 4.3. Correlation Analysis between Immune Infiltration Cells and m6A/m5C Subtypes

We analyzed the mRNA expression of major histocompatibility complex (MHC) genes, including human leukocyte antigen (HLA) genes, and immune checkpoint genes, to investigate the immune activity of the two clusters. The immune cells infiltration of cluster1 and cluster2 was estimated using the ESTIMATE algorithm [31], MCPcounter algorithm [32], and CIBERSORT algorithm [33,34] based on the gene expression profiles of 402 early-stage LUAD patients. Furthermore, chemokine genes and chemokine receptor genes expression and cytolytic immune activity (CYT) scores were analyzed to explore the relationship between m6A/m5C subtypes and immunity characteristics.

### 4.4. Somatic Mutation and Copy Number Alteration Analysis

We carried out somatic mutation and CNA analysis to explore the different genomic variations of cluster1 and cluster2. The mutation dataset was downloaded from the cBioPortal online tool (https://www.cbioportal.org/study/summary?id=luad_tcga, accessed on 20 October 2022) [31]. To reduce the false positive rate, we only retained non-synonymous mutations, such as the missense mutation, nonsense mutation, Frame_Shift_Del, Frame_Shift_Ins, In_Frame_Del, and In_Frame_Ins. The mutation type and frequency of the top mutant genes in each subtype were analyzed using the maftools R package and were visualized by a waterfall map [32]. TMB was defined as the total number of non-synonymous mutations in the coding region per trillion bases. To detect the signature of early-stage LUAD, we used 30 signature catalogs referenced in cosmic (http://cancer.sanger.ac.uk/cosmic, accessed on 22 October 2022). Regarding CNA data, a segment mean greater than 0.2 was defined as an amplification, less than −0.2 was defined as deletions, and CNA burden was defined as a change in gene copy number.

### 4.5. Differentially Expressed Genes (DEGs), Enrichment Analysis, and Selection Hub Genes of m6A/m5C Subtypes

The differentially expressed genes (DEGs) in cluster1 and cluster2 were analyzed by the ‘limma’ R package [33], and the threshold values were the false discovery rate (FDR) < 0.05 and | logFC | > 1. The potential functions of cluster-related differential genes were ascertained through Gene Ontology (GO) annotation and Kyoto Encyclopedia of Genes and Genomes (KEGG) enrichment pathway analysis by using the ‘clusterProfiler’ package [34]. Gene set enrichment analysis (GSEA) was performed using the R ‘clusterProfiler’ package [35], and gene set variation analysis (GSVA) was performed using the R ‘gsva’ package [36]. The reference gene set ‘c2.cp.kegg.v7.5.1.symbols.gmt’ was downloaded for GSEA and GSVA from MSigDB v7.5 to determine the statistical significance of the molecular pathway and the consistent heterogeneity between cluster1 and cluster2 [37]. We found the essential genes responsible for early-stage LUAD growth inferred by the CRISPR-based genome-wide loss-of-function screening analysis available at the Dependency Map portal (DepMap, https://depmap.org/portal/, accessed on 9 September 2022) [38]. Survival analyses were performed and validated in the GEO datasets.

### 4.6. Construction of the m6A/m5C-Related Risk Score

The best predictive biomarkers of m6A/m5C regulated genes were evaluated using the ‘glm net’ R package [39], and the least absolute shrinkage and selection operator (LASSO) Cox regression model [40,41]. In addition, the potential prognosis and biological mechanism of m6A/m5C regulatory genes were explored. We used the TCGA early-stage LUAD cohort as a training set, and GSE72094 and GSE50081 datasets as independent validation cohorts. Finally, seven m6A/m5C regulatory genes were screened out following the criteria of *p* < 0.05. The risk score formula was established as follows:

$$Risk\;Score=\sum_{i=1}^{n}\left(Coefficient\;of\;gene\;\left(i\right)\times Expression\;of\;gene\;(i)\right)$$

The coefficient of the gene (i) is the regression coefficient of gene (i), and the expression of the gene (i) is the expression value of gene (i) for each patient. Patients were divided into high-risk and low-risk groups according to the median risk score. In addition, the ‘survival’ R package was used to evaluate the OS difference between the high-risk and the low-risk group. The ROC curves were drawn using the ‘timeRoc’ R package [42] to measure the specificity and sensitivity of the predictive model.

### 4.7. Reverse Transcription-Quantitative Polymerase Chain Reaction (RT-qPCR)

The total RNA from 16 paired frozen fresh tumor tissues and paracancerous tissues of LUAD was extracted using the Maxwell^®^ RSC simply RNA Tissue Kit RNA (Promega, cat: AS1340) following the manufacturer’s protocol. According to the manufacturer’s instructions, the reverse transcription of RNA to cDNA was obtained using the HiScript^®^ III All-in-one RT SuperMix Perfect for qPCR (Vazyme, Cat: R333-01). The qPCR was performed with a StepOne™ Real-Time PCR System (Thermo Fisher, Waltham, MA, USA) in a 20 μL reaction mixture containing SYBR Greene. The expression of different genes was normalized to GAPDH and was analyzed using the 2^−(∆∆Ct)^ method [43]. (ΔCT = CT (target gene) – CT (reference gene), ΔΔCT = ΔCT(tumor sample) – ΔCT(normal sample)). The expression levels between normal and tumor tissues were compared using the Wilcoxon test.

### 4.8. Statistical Analysis

All statistical analyses were carried out utilizing R software (version 4.2.1). The chi-square test analyzed the relationship between clinical variables and subtypes. The correlation coefficient was calculated using Pearson correlation analysis. The Wilcoxon test was used to investigate the difference between the groups. The R ‘ggplot2′ packages were used for data visualization. The KM curve and log-rank test were used to evaluate differences in survival rate between the groups. All survival curves were generated using the ‘survival’ [44] and ‘survminer’ [45] R packages. All *p*-values were two-sided, and *p* < 0.05 was considered statistically significant.

## Figures and Tables

**Table 1:** RT-qPCR primer sequences were used in this study.

Primers	Sequences	
METTL3	Forward	ACAGGCCGTACAGGTCAC
	Reverse	ATCACATCACAATCCAGACCCT
NPLOC4	Forward	CCTCGTCTCAGAAGATACCCGAA
	Reverse	TTGGGATGCTTGTTCTGGAAGTC
NSUN3	Forward	TGCCCAGCCTGAAATGTTTG
	Reverse	TGAGAGTCAGAAGAAAACAACCAG
NSUN7	Forward	GCAGCATTGGCAAGATGTCGA
	Reverse	TGGAGGCCCTTAGTTCCTGT
RBM15	Forward	GGCTGCCTGAGGAGAGTGGAG
	Reverse	CGGCTACTGCTCAATTCTGGACTG
YTHDF1	Forward	CCCAGAGAACAAAAGGACAAGA
	Reverse	TGTCCAGTAAGGTAGGGCTCAA
IGF2BP1	Forward	ATGGTTATCATCACTGGACCG
	Reverse	TGTGGGTCTCCAGCTTCACT
GAPDH	Forward	TGCACCACCAACTGCTTAGC
	Reverse	GGCATGGACTGTGGTCATGAG

## Data Availability

The original cohorts data presented in the study are included in the Supplementary Tables. More information is available upon reasonable request from the corresponding author.

## Supplementary Materials

The following supporting information can be downloaded at: https://www.mdpi.com/article/10.3390/ijms24076520/s1.
