# Supplementary material for: Prognostic Value and Genome Signature of m6A/m5C Regulated Genes in Early-Stage Lung Adenocarcinoma
Source: Int J Mol Sci. 2023 Mar 30;24(7):6520. doi: 10.3390/ijms24076520 (PMC10095361; doi:10.3390/ijms24076520)
Supplement: Supplementary file 1 [file ijms-24-06520-s001.zip › Supplementary Figures.pdf]

Supplementary Figures

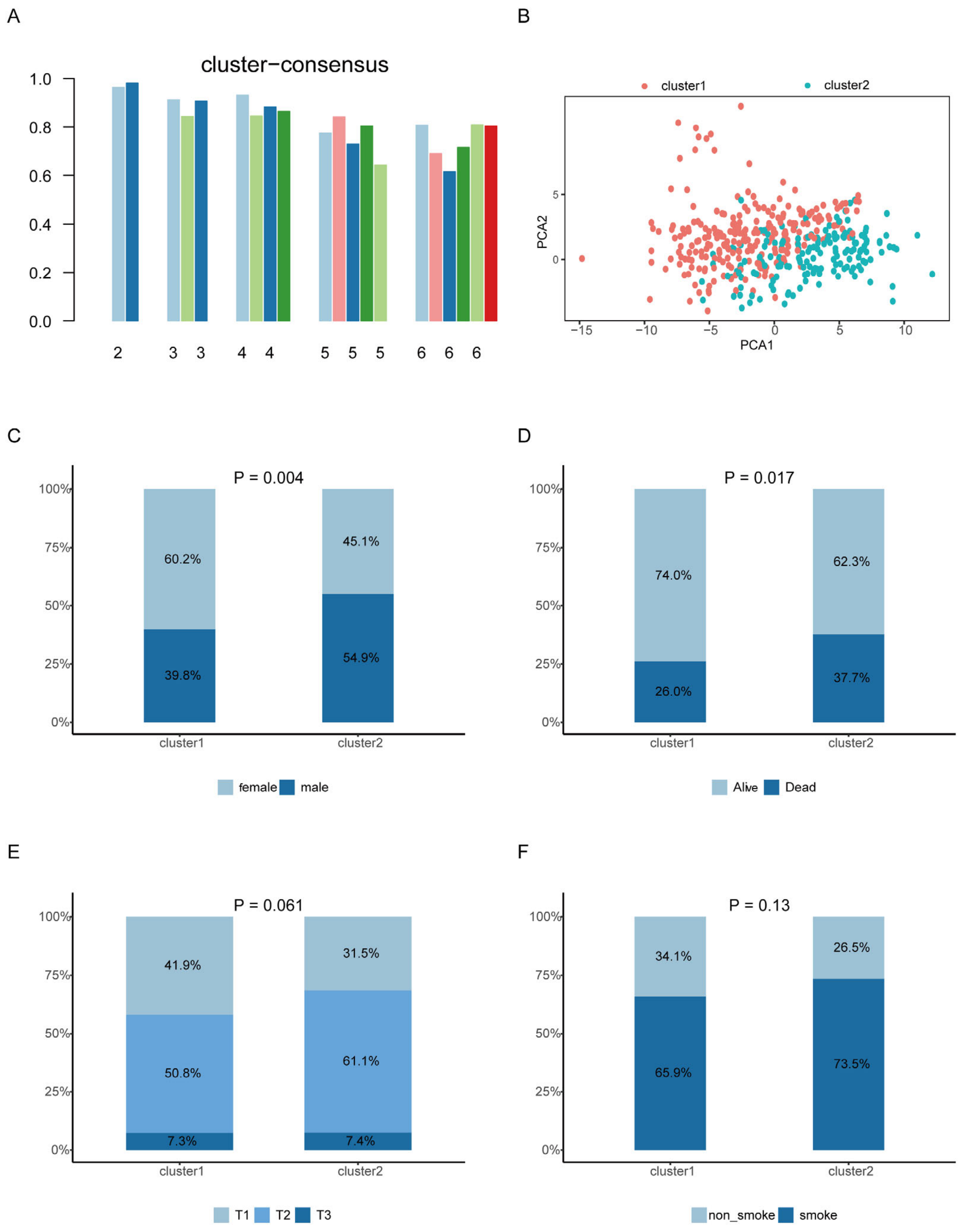

Figure S1 Detail result of clustering, PCA and clinical features between molecular clusters. (A) The Cluster-Consensus Plot represented the chosen optimal cluster number (k= 2) for m6A/m5C genes. (B) PCA analysis of m6A/m5C clusters in TCGA cohort. (C-F) Comparison of the distribution of different clinical features between the two m6A/m5C clusters in TCGA dataset.

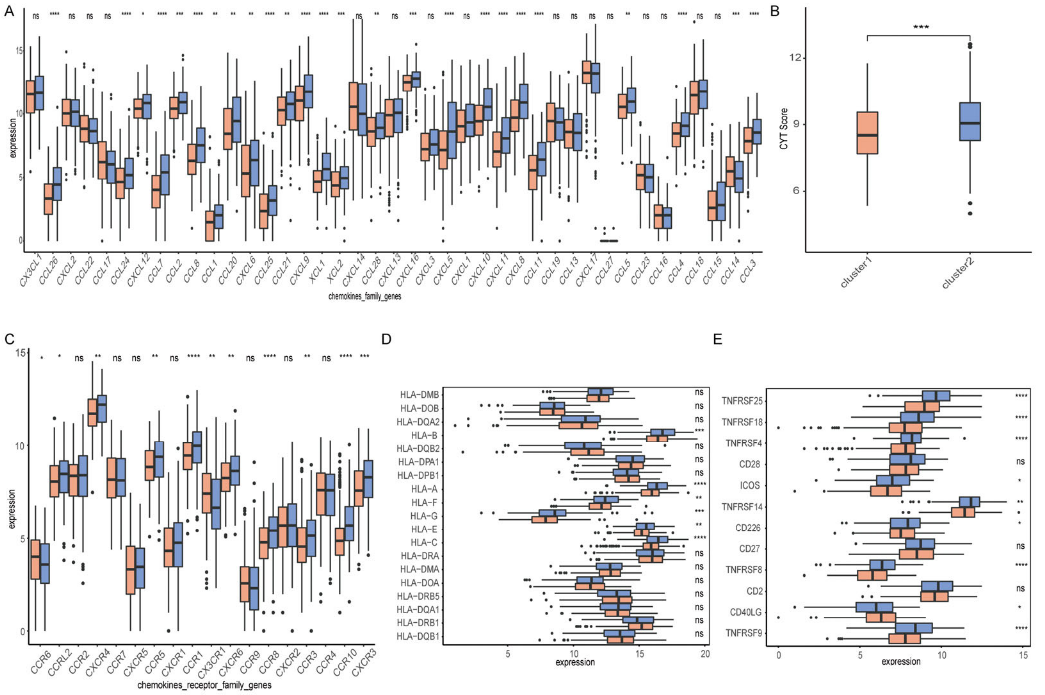

Figure S2 Expression of chemokine and MHC in different m6A/m5C subtypes.(A) The expression of chemokine genes in m6A/m5C subtypes. (B) The score of Cytolytic activity in m6A/m5C subtypes. (C) The expression of chemokine receptors genes in m6A/m5C subtypes. (D) Gene expression of HLA gene sets between the m6A/m5C clusters. (E) Gene expression of MHC gene sets between the m6A/m5C clusters. Statistical significance at the level of ns ≥ 0.05, * <0.05, ** <0.01, *** <0.001 and **** <0.0001.

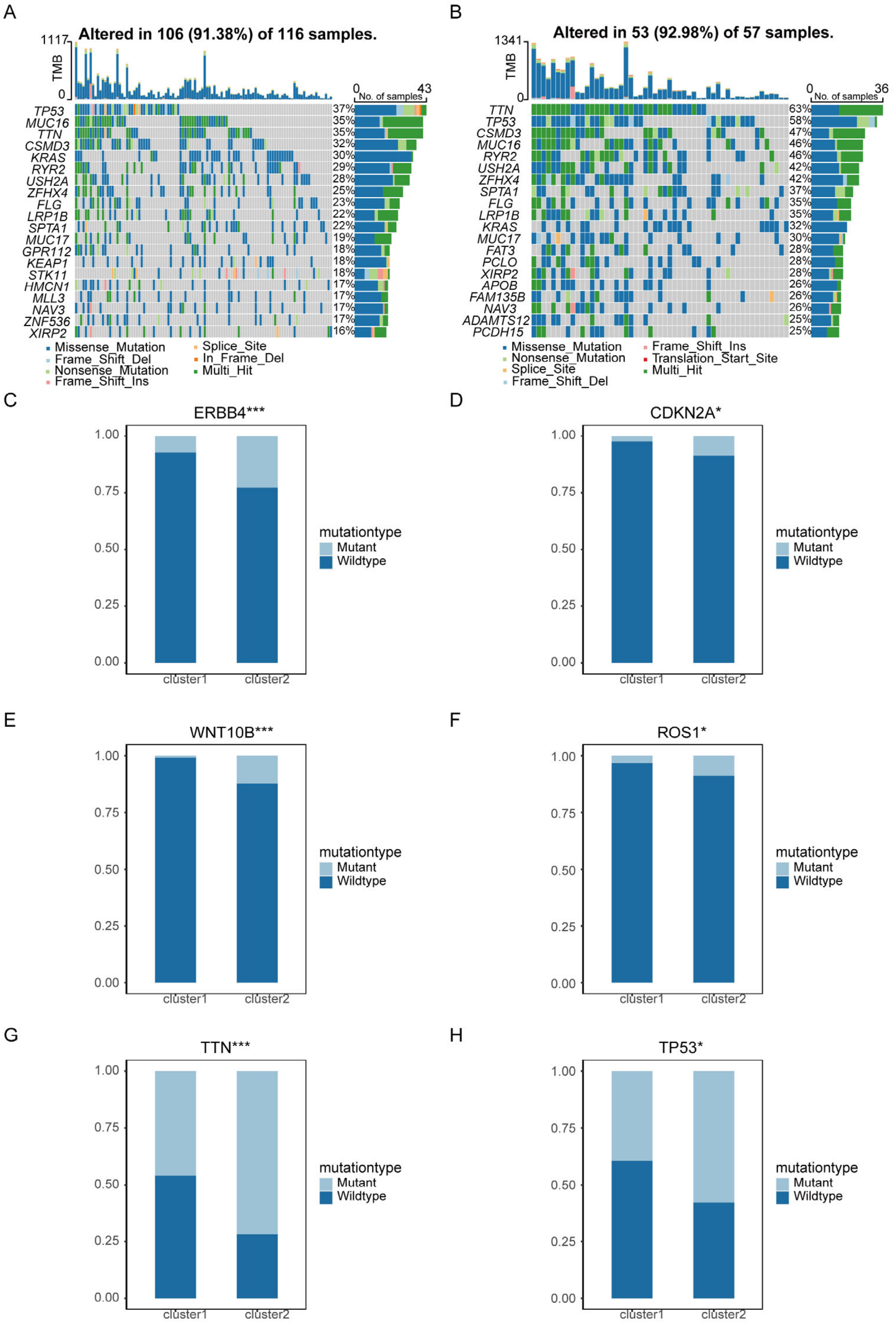

Figure S3 Detailed results of mutation analysis between cluster1 and cluster2. The waterfall plots showing the top 20 mutated gene of cluster1(A)and cluster2(B). (C-H) Barplots showing the mutation frequency of 6 differently mutating genes in clusters.

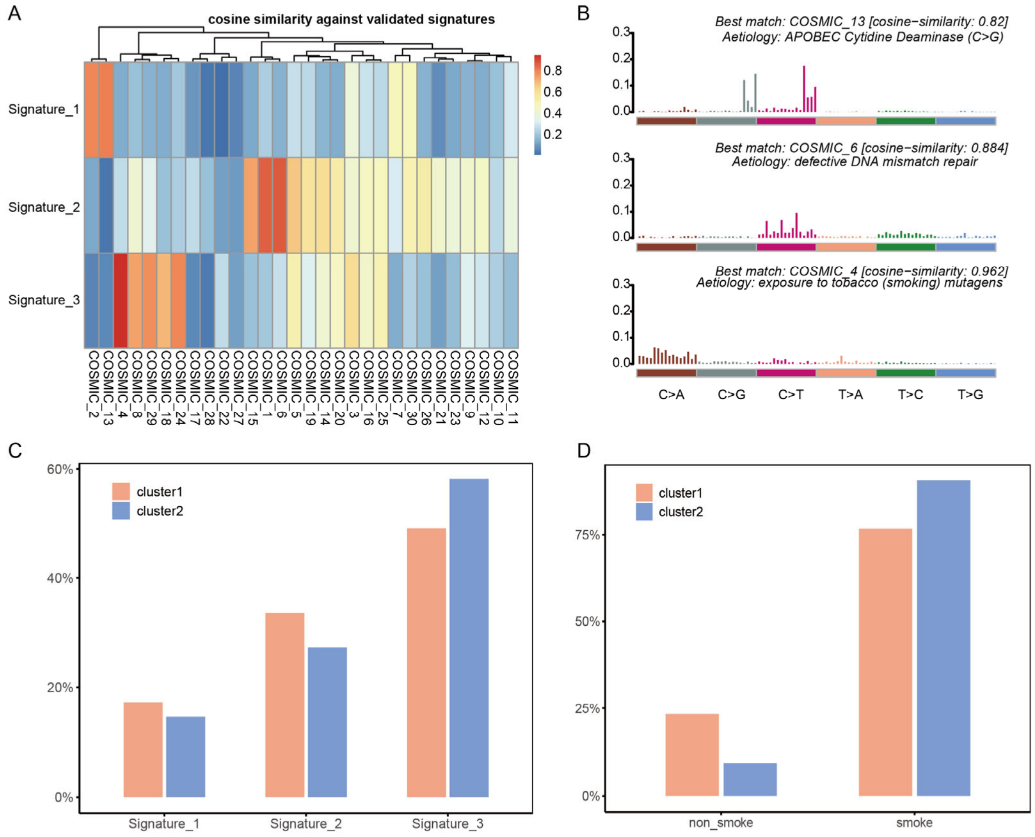

Figure S4 Mutation signature analysis of the early-stage TCGA-LUAD.(A-B) Mutational signatures associated with the early-stage TCGA-LUAD. (C) Comparison of the distribution of m6A/m5C subtypes among the three mutational signatures. (D) Comparison of the distribution of m6A/m5C subtypes between different smoking status in signature 3.

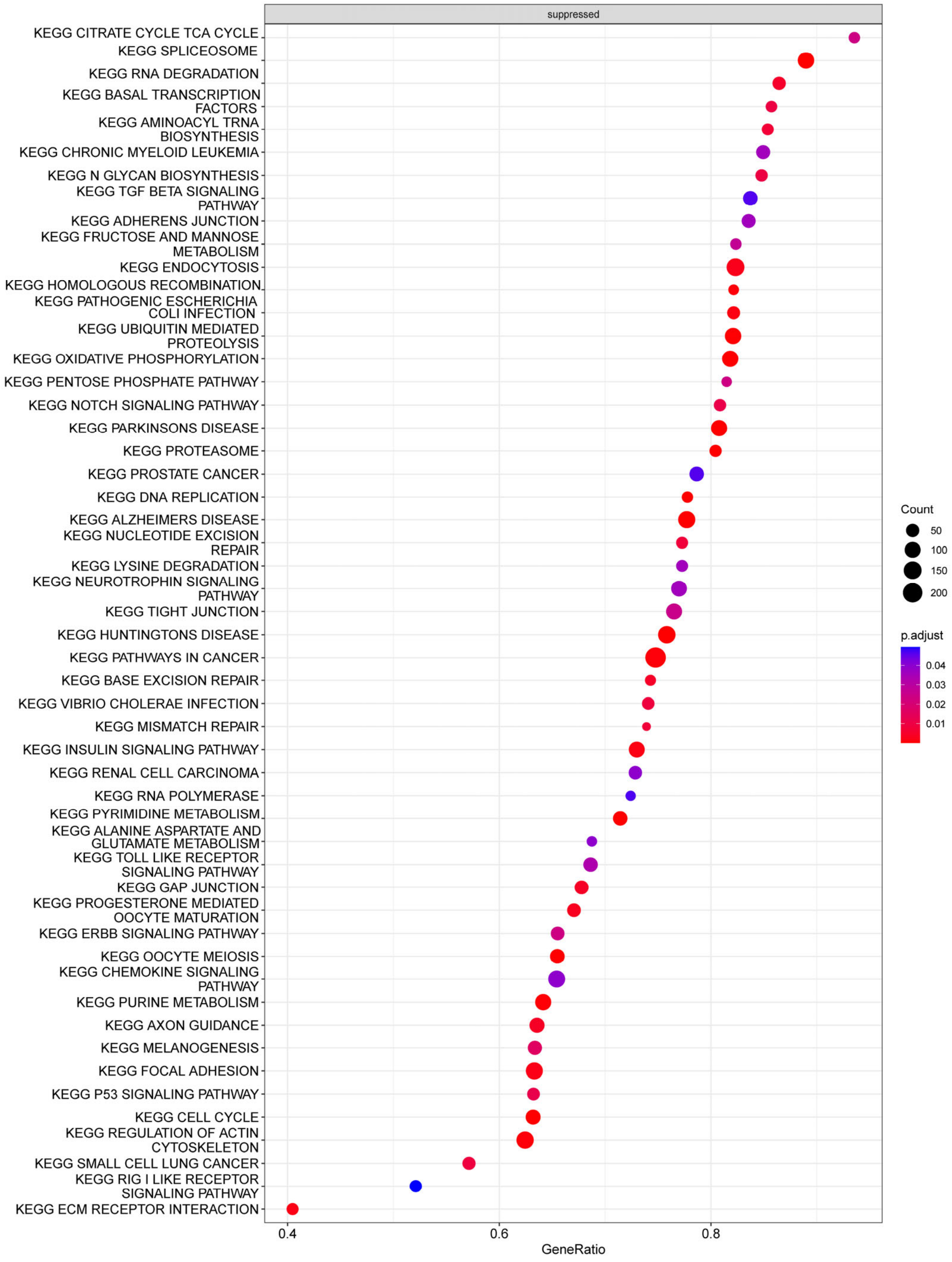

Figure S5 The result of GSEA between the cluster1 and the cluster2.

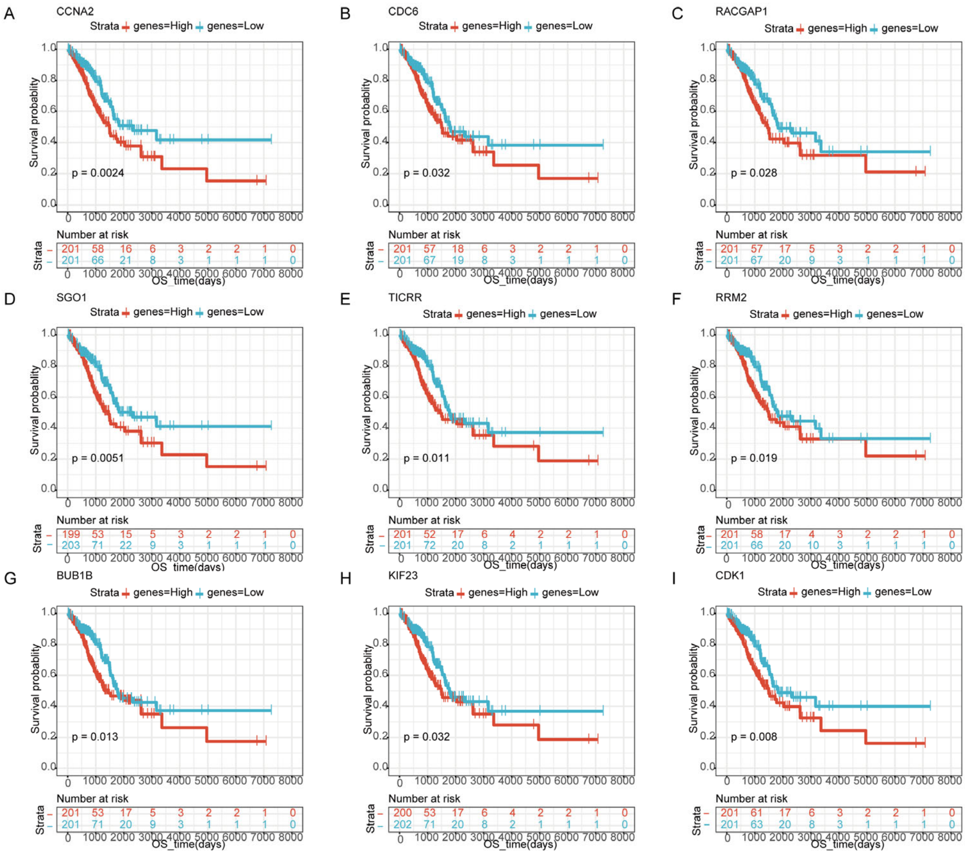

Figure S6 Detailed results of survival analysis (Kaplan–Meier) for OS in early-stage TCGA-LUAD. (A-I) Kaplan–Meier analysis shows the association between the expression of the 9 hub gene and early-stage LUAD patient overall survival (OS) in TCGA cohort.

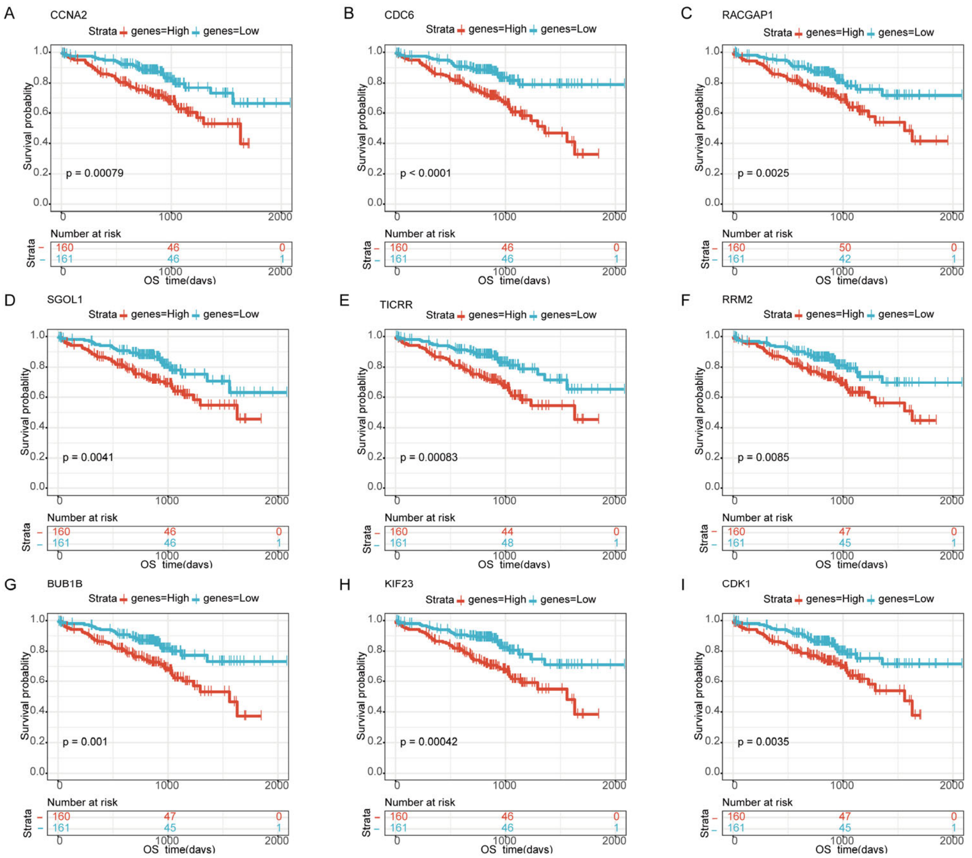

Figure S7 Detailed results of survival analysis (Kaplan–Meier) for OS in GSE72094. (A-I) Kaplan–Meier analysis shows the association between the expression of the 9 hub gene and early-stage LUAD patient overall survival (OS) in GSE72094.

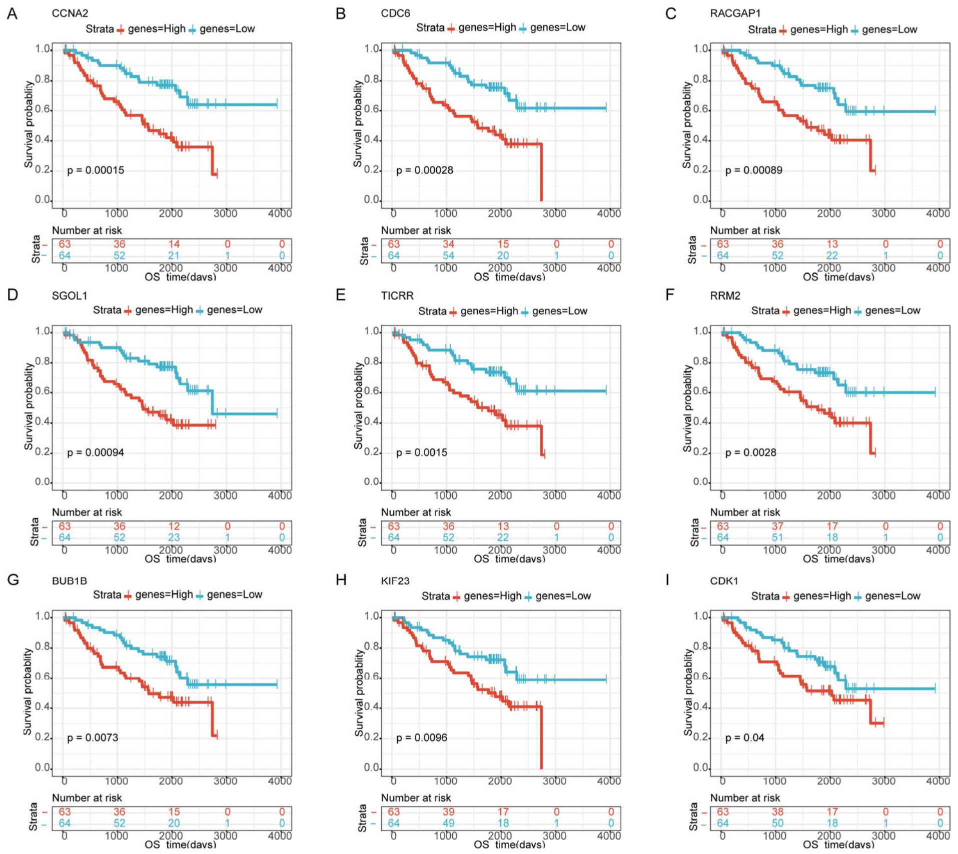

Figure S8 Detailed results of survival analysis (Kaplan–Meier) for OS in GSE50081.(A-I) Kaplan–Meier analysis showing the association between the expression of 9 hub gene and early-stage LUAD patient overall survival (OS) in GSE50081.

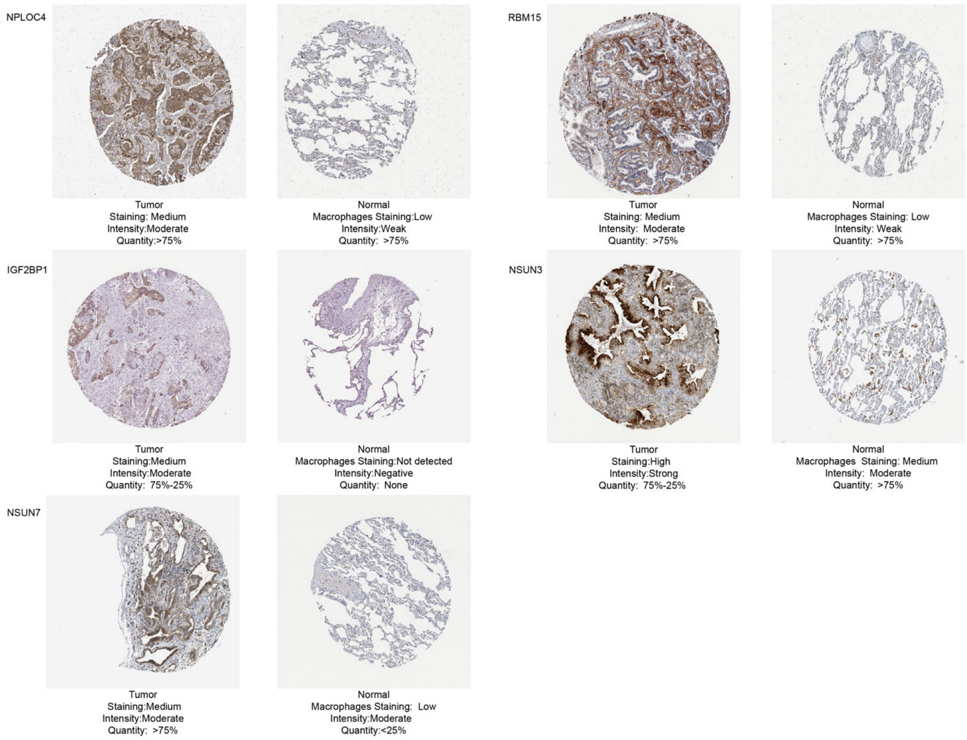

Figure S9 The protein expression of 5 prognostic genes (NPLOC4, RBM15, IGF2BP1, NSUN3, and NSUN7) in immunohistochemical images of lung tumor (left) and normal(right) groups.
